# Calmodulin and Its Interactive Proteins Participate in Regulating the Explosive Growth of *Alexandrium pacificum* (Dinoflagellate)

**DOI:** 10.3390/ijms23010145

**Published:** 2021-12-23

**Authors:** Yuan Liu, Zhimei Zhu, Zhenghong Sui, Haoxin Liu, Sadaf Riaz

**Affiliations:** 1 Key Laboratory of Marine Genetics and Breeding of Ministry of Education of China, College of Marine Life Sciences, Ocean University of China, Qingdao 266003, China; liuyuan_1991@yeah.net (Y.L.); zhimeizhu@163.com (Z.Z.); lhx@stu.ouc.edu.cn (H.L.); sadafriaz.microbiology@gmail.com (S.R.); 2 College of Agronomy, Qingdao Agricultural University, Qingdao 266109, China; 3 Shenzhen Graduate School, Peking University, Shenzhen 518055, China

**Keywords:** harmful algal blooms, *Alexandrium pacificum*, calmodulin, cell cycle, 2-D analysis, far western blot

## Abstract

*Alexandrium pacificum* is a typical dinoflagellate that can cause harmful algal blooms, resulting in negative impacts on ecology and human health. The calcium (Ca^2+^) signal transduction pathway plays an important role in cell proliferation. Calmodulin (CaM) and CaM-related proteins are the main cellular Ca^2+^ sensors, and can act as an intermediate in the Ca^2+^ signal transduction pathway. In this study, the proteins that interacted with CaM of *A. pacificum* were screened by two-dimensional electrophoresis analysis and far western blots under different growth conditions including lag phase and high phosphorus and manganese induced log phase (HPM). The interactive proteins were then identified using matrix-assisted laser desorption ionization time-of-flight mass spectrometry. Four proteins were identified, including Ca^2+^/CaM-dependent protein kinase, serine/threonine kinase, annexin, and inositol-3-phosphate synthase, which all showed high expression levels under HPM. The gene expression levels encoding these four proteins were also up-regulated under HPM, as revealed by quantitative polymerase chain reaction, suggesting that the identified proteins participate in the Ca^2+^ transport channel and cell cycle regulation to promote cell division. A network of proteins interacting with CaM and their target proteins involved in the regulation of cell proliferation was raised, which provided new insights into the mechanisms behind the explosive growth of *A. pacificum*.

## 1. Introduction

Harmful algal blooms (HABs) have become one of the main oceanic disasters. In the past few decades, their intensity and frequency have increased significantly, especially those caused by dinoflagellates, and have attracted public concern [1]. Dinoflagellates of the genus *Alexandrium* are causative agents of HABs in the coastal zone. They can produce toxins that are responsible for marine fish and shellfish mortality. Moreover, they can affect human health via the ingestion of toxins from seafood [2,3,4]. Many recent studies have investigated the physical processes and biological mechanisms involved in HABs [5,6,7,8]. The concentrations of nutrients and some special organic substances in water directly affect the growth of red tide organisms [9,10]. Physiological and ecological studies showed that different nitrogen and phosphorus concentrations and microelements (iron and manganese) would affect the growth rate and cell density of dinoflagellate [11]. The increase of phosphorus concentration promoted the growth of dinoflagellate [12]. Manganese is a component of oxygen evolution complex (OEC) and plays an important role in algae growth and photosynthesis; and the lack or increase of manganese will inhibit or promote the growth of algae in varying degrees [13,14,15]. The molecular mechanisms involving algal cell division and growth regulation are critical to the formation of HABs; however, the respective research is limited.

The calcium (Ca^2+^) signal transduction pathway plays an important role in many eukaryotic cell processes, such as biotic and abiotic stress-responses and cell proliferation. Calmodulin (CaM) and CaM-related proteins are the main cellular Ca^2+^ sensors that can act as an intermedium in the signal transduction pathway [16,17]. In the past several years, our understanding of how Ca^2+^/CaM regulate cell cycle transitions has advanced. In the root tip cells of plants, CaM levels increase two to four folds at the G1/S phase boundary, and the inhibition of CaM can interrupt DNA synthesis and arrest the G1 cell cycle phase through blocking the phosphorylation of calcineurin (serine/threonine kinase) [18]. At the transition from the G2 to M phase, CaM levels increase rapidly in *Aspergillus nidulans* [19]. Repression of CaM expression using the alcA promoter can arrest the majority of cells in G2 [20]. In marine dinoflagellates, CaM and other calcium-related genes play important roles in response to heat stress and calcium regulation, including calcium binding and transport, which has been reported recently [21]. The CaM sequence showed high conservation, with 97.2–98.3% homology to that of *Alexandrium fundyense*, *Pfiesteria piscicide*, and *Karlodinium micrum* in dinoflagellates, 93.1–95.2% homology to that of other algae, such as *Saccharina japonica* and *Thalassiosira pseudonana*, with 91.3–92.6% homology to that of high plant and animals [22], which indicated CaM of dinoflagellates have the similar functions.

Ca^2+^/CaM -dependent protein kinase II (CaMKII), the target enzyme of CaM, regulates the transition from the G2 to M phase through activating the cell division cycle (cdc) 2/cyclin B gene [23]. In yeast, cells accelerate the entry into the M phase through inducing the expression level of CaM. The inhibition of CaMKII activity can lead to the stagnation of mitosis via blocking the Ca^2+^/CaM pathway [24,25]. In *Saccharomyces cerevisiae*, when the expression of the CaM gene was modulated manually, repression of CaM resulted in nuclear division defects with cells having short mitotic spindles and increased chromosomal loss [26,27]. Morse et al. (2016) found that most yeast cell cycle regulators have homologs in these dinoflagellates, suggesting that the yeast model is appropriate for understanding regulation of the dinoflagellate cell cycle [28].

Dinoflagellates follow a typical eukaryotic G1-S-G2-M cell cycle, and cyclin-dependent kinase (CDK)-like proteins or genes have also been found in dinoflagellates [29]. A cyclin B-like protein in *Karenia brevis* may regulate cell cycle progression from the G2 phase to the M phase [30]. Cdc2- like kinase has also been detected in *Gambierdiscus toxicus*, and its expression was tracked through the cell cycle [31]. The interactions of cyclins and cdc kinase drive cell cycles in most eukaryotic organisms [32]. Studies investigating cyclin and cdc kinase in dinoflagellates have suggested that dinoflagellates may possess the same cell cycle mechanisms. However, the specific cell cycle progression remains unknown due to the specific features, enormous genomes in liquid crystal states, of dinoflagellates [33]. In addition, the Ca^2+^/CaM pathway is highly conserved among different organisms, which suggests that it may play the same role in dinoflagellates [16].

For unicellular marine phytoplankton, cell cycle progression is critical to their growth. Therefore, research into cell cycle progression and its regulation are important for revealing the mechanisms of dinoflagellate bloom formations. Due to the critical roles of Ca^2+^/CaM signal pathways in the cell cycle, studying the proteins that interact with CaM can help determine the influence of the Ca^2+^/CaM signal pathway on the cell cycle [17,34]. Furthermore, it will provide insight into the cell cycle mechanisms in *Alexandrium pacificum.*

The outbreak of dinoflagellate blooms includes four distinct phases: initiation, development, maintenance, and dissipation. In the development (LOG) phase, algal organisms proliferated rapidly induced by factors, such as temperature, light, and nutrients. The process from formation to extinction of a red tide is similar to the growth curve of single- celled microorganisms. As the final products of gene expression, a high dependency and specificity of proteins were found to vary with growth phases [35]. Liu et al. (2006) discovered that the effect of different nutrient supply on the growth of *P. donghaiense* Lu involved the Ca^2+^/CaM signal transduction pathway, which suggested that nutrient can regulate marine phytoplankton interaction with the environment through Ca^2+^/CaM signal pathway [36]. The full cDNA sequence of the *cam* gene in *A. pacificum* has been cloned. The abundance of *cam* transcript varied in a pattern similar to cell growth rate during the whole growing period [22]. The *cam* transcription abundance increases more than eight-fold from the lag phase to the exponential phase [22]. The *cam* gene was also found to be upregulated during the explosive growth process of *A. pacificum* via a digital gene expression profiling analysis [36].

In this study, *A. pacificum* was cultured and collected under three conditions, including the lag phase (LAG), log phase (LOG), and high phosphorus- and high manganese-induced log phase (HPM), which aimed to simulate the conditions before and within the process of HABs. Far western blot technology was used to screen the proteins that interacted with CaM under LAG and HPM. In order to detect differences in proteins after treatment, two-dimensional(2-D) difference gel electrophoresis, a high-resolution gel-based quantitative protein method, was used [37]. The differentially expressed proteins were identified using matrix-assisted laser desorption ionization time-of-flight mass spectrometry (MALDI-TOF MS). Quantitative real-time polymerase chain reaction (qPCR) was then used to record the gene expression of the differentially expressed proteins under the transcription level of the three conditions mentioned above. The aim of this study was to investigate the mechanisms of CaM and its interactive proteins underlying the cell cycle and cell proliferation regulation of *A. pacificum*.

## 2. Results

### 2.1. Heterologous Expression in E. coli and GST-CaM Fusion Protein Purification

**Figure 1:**
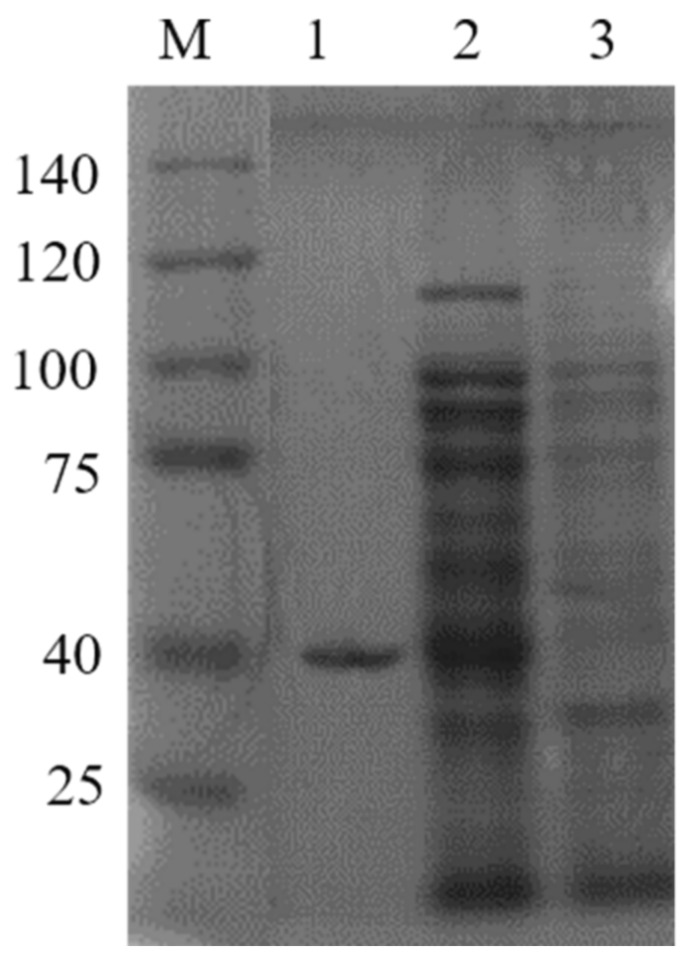
SDS-PAGE analysis of prokaryotic expressed and purified GST-CaM fusion proteins. Line 1: purified GST-CaM fusion protein; line 2: ultrasound supernatant of GST-CaM/BL21 (DE3); and line 3: ultrasound precipitation of GST-CaM/BL21 (DE3).

The *cam* coding sequence from *Alexandrium pacificum* was subcloned into a PGEX-6P-1 vector. After induction with 1 mM IPTG for 8 h at 28 °C, SDS- PAGE results showed that both the supernatant and precipitate contained the target protein; however, the supernatant had a larger amount of target protein than the precipitate, indicating that the recombinant proteins were mainly expressed in a soluble form. The GST-CaM fusion protein was then purified using glutathione-sepharose 4B resin, and the band was approximately 40 kDa, which is consistent with the theoretical value of GST-CaM fusion protein (Figure 1).

### 2.2. Determination of GST-CaM Specificity

**Figure 2:**
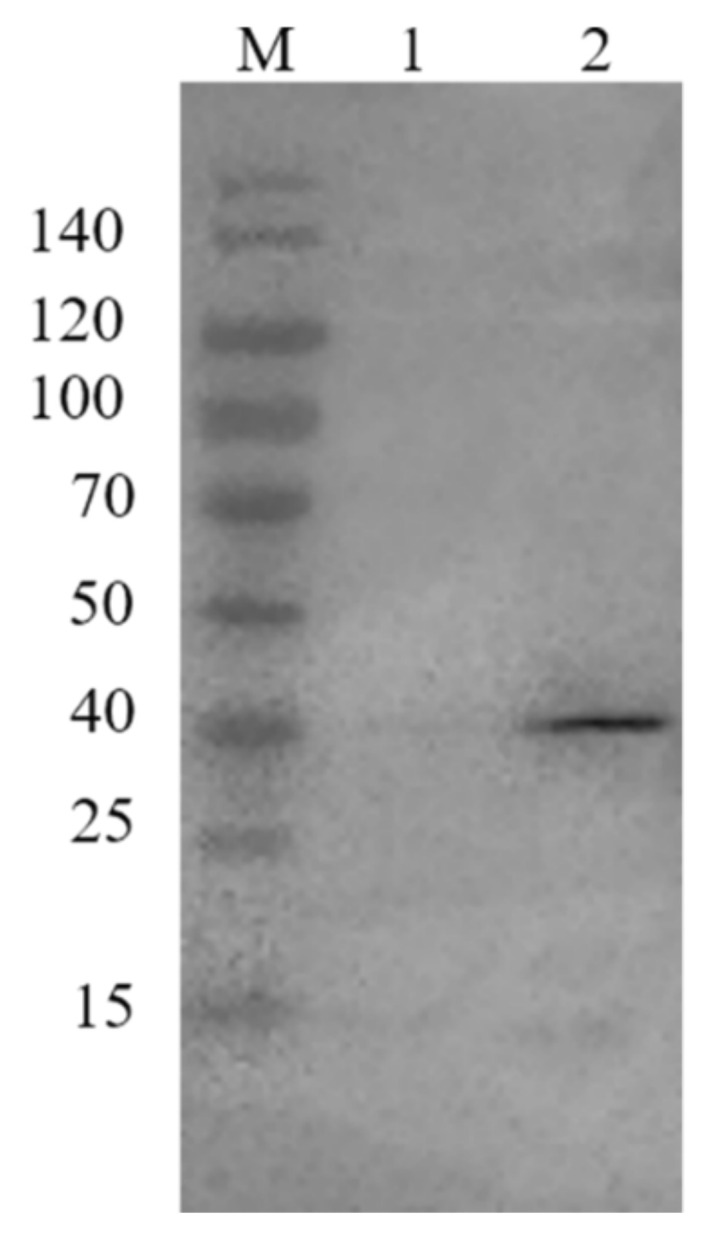
Confirmation of GST-CaM specificity with anti-GST antibody. Line 1: control with the pre-immune serum; line 2: anti-GST antibody reacted with GST-CaM fusion protein.

To determine the specificity of GST-CaM, the anti-GST antibody was used to detect the purified GST-CaM fusion protein from the supernatant by Western Blot. The antibody was specifically affined to a 40 kDa protein band, which confirmed that GST-CaM was successfully expressed with purified homogeneity (Figure 2).

### 2.3. 2-D and Far Western Blot Analysis of Differential Protein Expression

**Figure 3:**
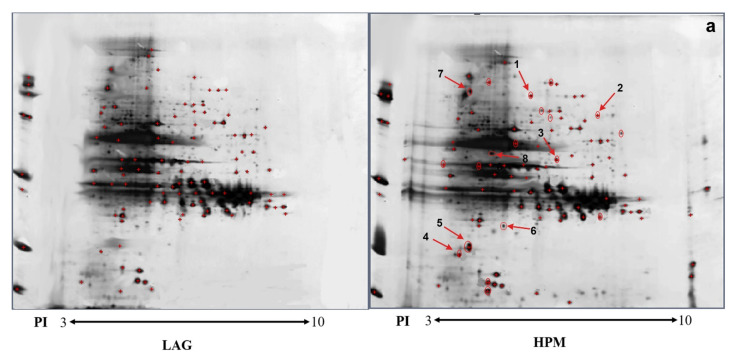

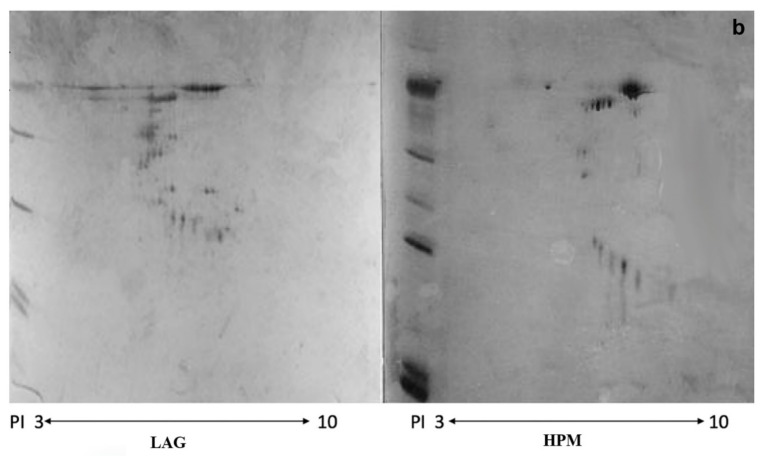
Two-dimensional analysis of protein extracts of *A. pacificum* at different growth phases with GST as the control. (**a**): No.1–4 spots represent proteins only present in HPM. No. 5–8 represent the top four ranked differential expressed spots whose expression in HPM are significantly up-regulated compared with LAG; (**b**): GST as a control for the far western blot and 2-D analysis.

Total proteins under the LAG and HPM of *A. pacificum* were extracted and analyzed in 2-D. The proteins that interact with GST-CaM and GST (as control) were then screened by far western blot, respectively. Protein spots were detected by PDQuest software (Figure 3). A total of 98 protein spots were common under the two conditions, and 17 protein spots exhibited a statistically significant difference between the two conditions (*p* < 0.05), with the variations in abundance exceeding two-fold. Four differentially expressed protein spots, which were only present in HPM, and the top four-ranked differential spots, whose expression in HPM were significantly up-regulated compared to LAG, were submitted for identification using MALDI-TOF MS and were searched in the Swiss-Prot, National Center for Biotechnology Information Non-redundant protein database. Four positive identifications were obtained, including two identifications that were only present in HPM. The detailed information of identified proteins is listed in Table 1. The positive identifications included Ca^2+^/CaM -dependent protein kinase 2B (CaMK2B), STK, annexin A4, and inositol-3-phosphate synthase (MIPS3).

### 2.4. Gene Expression Analysis of Differentially Expressed Proteins

A standard curve was established to calculate the amplification efficiency of CaMK2B, STK, MIPS3, annexin, actin, and GAPDH, respectively. A good linear correlation was demonstrated in every standard curve for the qPCR assay with a correlation coefficient greater than 0.98. According to the formula E = 10^(1/slope) − 1, the amplification efficiency of the four genes was 102%, 104%, 104%, and 104%, respectively, and the melting curves showed a single peak, corresponding to a single sized transcript for each gene. Therefore, the cycle threshold value could be used in the gene expression analysis.

**Figure 4:**
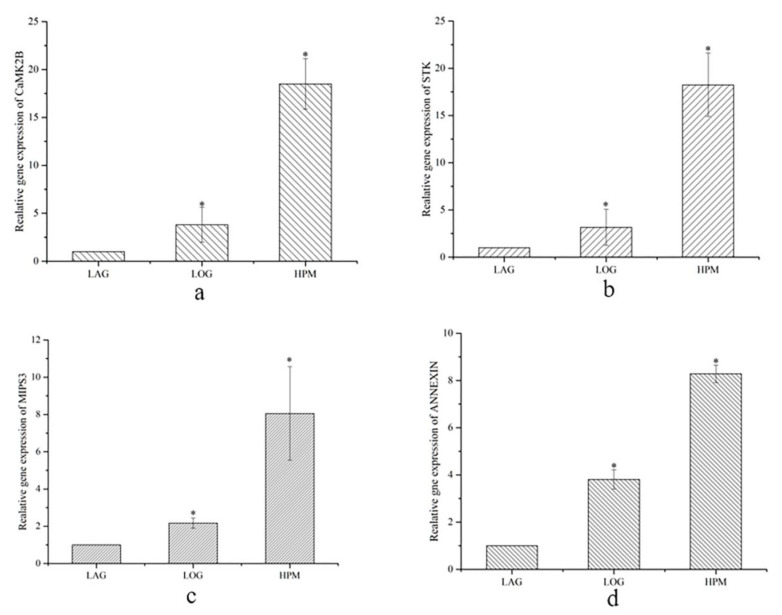
Relative gene expression of the differential expressed proteins identified by MS. (**a**): CaMK2B; (**b**): STK; (**c**): MIPS3; (**d**): ANNEXIN. Expression levels of CaMK2B, STK, MIPS3, and ANNEXIN were examined by quantitative RT -qPCR. The *Alexandrium pacificum* housekeeping gene actin and GAPDH were used as internal reference. The relative gene expression level is the ratio of each gene expression in LOG and HPM relative to LAG. The symbol * represents a significant difference.

**Figure 5:**
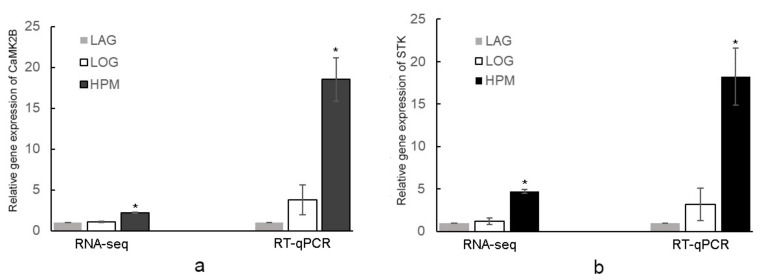
Relative expression of CaMK2B and STK under different growth phases of *A. pacificum* from RNA-seq and RT-qPCR. (**a**): CaMK2B; (**b**): STK. The relative gene expression level is the ratio of each gene expression in LOG and HPM relative to LAG. The symbol * represents a significant difference.

Normalized gene expression according to the 2^−∆∆CT^ method is shown in Figure 4. Expression of the four genes in LOG and HPM was higher than those in LAG (*p* < 0.05). In LOG and HPM, the relative expression levels of CaMK2B, STK, MIPS3, and annexin were 3.81 (in LOG) and 18.51 (in HPM); 3.16 (in LOG) and 18.25 (in HPM); 2.17 (in LOG) and 8.06 (in HPM); and 3.81(in LOG) and 8.28 (in HPM), respectively. Moreover, the expression of gene encoding CaMK2B and STK was also analyzed from the transcriptome data (SRX368254), and the expression of CaMK2B and STK in LOG and HPM was also higher than those in LAG (Figure 5).

## 3. Discussion

### 3.1. Ca^2+^ Transport Channel

In all eukaryotic cells, Ca^2+^ is required in both the extracellular environment and intracellular stores for cell growth and division [38,39]. The transient increase in the concentration of free Ca^2+^ in the cytosol and its spread to the nucleus lead to cell activation, which is involved in the binding of a broad range of stimuli including mitogenic factors and other transcription factors, and further initiates many signal transduction processes [40,41]. The transport systems that control the concentration of Ca^2+^ in the nucleus are of great importance for the cell.

The endoplasmic reticulum (ER) serves as a major intracellular Ca^2+^ store. Inositol 1,4,5-trisphosphate (IP3) is an important messenger for Ca^2+^ signals downstream of G protein-coupled receptors and receptor tyrosine kinases [42]. Inositol trisphosphate receptor is a membrane glycoprotein complex acting as a Ca^2+^ channel activated by IP3. Myo-inositol (MI) is the precursor of phosphatidylinositol 4,5-bisphosphate (PIP2), which can be catalyzed by phospholipase C (PLC) to produce IP3. In the biosynthesis of MI, the rate-limiting step is the conversion of D-glucose-6-P to MI-1-P, which is catalyzed by MIPS3 [43]. Depletion of intracellular IP3-sensitive Ca^2+^ stores with pharmacological interventions, such as thapsigargin or 2,5-di-*tert*-butyl- hydroquinone, results in a cessation of cell division [44]. These agents block the Ca^2+^ pumping ATPase present in the ER and result in a depletion of Ca^2+^ stores in the ER. The consequences of intracellular Ca^2+^ pool depletion include inhibition of DNA synthesis, protein synthesis, and nuclear transport [45]. In this study, the expression of MIPS3 at both the gene and protein level was up-regulated under HPM suggesting that a large amount of MIPS3 was needed to guarantee the biosynthesis of IP3, which then activates the Ca^2+^ channel, and provides sufficient Ca^2+^ for the cell cycle.

Annexins are family members of proteins that bind acidic phospholipids in the presence of Ca^2+^ and are involved in Ca^2+^-dependent exocytosis [46,47]. Recent studies found that annexins possessed voltage-gated Ca^2+^ channel activity suggesting that they may insert into the phospholipid bilayer and work as ion channels to transport Ca^2+^ [48]. It has been proposed that annexins increase membrane permeability to Ca^2+^ by two distinct mechanisms, which depend on the concentration of annexin in the membrane and their aggregation state. At high annexin concentration, an intermolecular pore is formed by annexin hexamers; at lower concentration, permeation occurs is through the monomer.

Phosphatidylinositol 4,5-bisphosphate (PIP2) functions as an intermediate in the IP3 pathway and is catalyzed by PLC to produce IP3 and diacylglycerol (DAG), which is crucial for the regulation of the actin cytoskeleton. Annexin is a binding partner of PIP2 at the actin-rearrangement sites [49]. Because PIP2 is a precursor for IP3 and DAG, and it is involved in vesicular trafficking and cell motility; these results supported the role of annexin in the regulation of membrane cytoskeleton dynamics in vesicle trafficking and indicate a potential role in other cell signaling events [50].

Furthermore, the cellular content of annexin has also been shown to change during the cell cycle. As cells divide and enter the G1 phase there is a general decrease in the annexin concentration. New synthesis of annexin occurs as the cells enter the S phase; however, as the cell progresses through the S phase, there is a general reduction in the protein [48,51,52]. In this study, during the HPM of *A. pacificum*, the expression of annexin was up-regulated, both at the mRNA and protein level. The up-regulated annexin in *A. pacificum* not only transports sufficient Ca^2+^ for algal cells but also makes protein reserves for cells entering the S phase. Additionally, the interaction with CaM suggested that it may be regulated by the Ca^2+^/CaM pathway in transporting Ca^2+^ into the cell and may play a role in the cell cycle.

### 3.2. CaM and Its Interacting Protein in Cell Cycle Regulation

The cell cycle in eukaryotic organisms has been studied for decades; progression through the cell cycle requires several processes, such as DNA replication and chromosome separation [53,54]. However, the mechanisms of the dinoflagellate cell cycle remain unknown due to their unique features. The Ca^2+^/CaM pathway is essential in regulating the cell cycle and numerous intracellular proteins involved in a myriad of pathways. Therefore, identification of conserved Ca^2+^/CaM binding proteins that regulate cell cycle progression remains difficult [17]. In this study, the quantitative protein approach was used to compare the proteins that interact with CaM at different growth stages of *A. pacificum*.

The Ca^2+^/CaM pathway is essential in regulating the cell cycle. Previous research has suggested that the roles of CaM in cell proliferation and the control of the cell cycle are mediated by CaM-dependent phosphorylation/dephosphorylation events [55]. The best-studied kinases involved in these processes are the multifunctional Ca^2+^/CaM-dependent protein kinases (CaMKs) [56]. All types of CaMKs feature in the transcriptome data (SRX368254) of *A. pacificum* including CaMK I, CaMK II, CaMK III, and CaMK IV. Although all these kinases are regulated by phosphorylation, the mechanisms and functions differ among types. CaMK I functions in the cell cycle, especially in G0/G1 transition, CaMK IV participates in the phosphorylation of transcription factors, while CaMK III phosphorylates eEF2 during active cell proliferation.

CaMK II plays a critical role in activating the mitogen-activated protein kinase (MAPK) pathway and controlling cell cycle (G1/S and G2/M). At the G1/S transition, CaMK II may negatively regulate G1/S transition by phosphorylating the large subunit of the replication factor C, thus preventing DNA replication [57,58,59]. The potential role of CaMKII during G1/S is centrosome duplication. During G2/M transition, the cell division cycle protein, cdc25C is activated by phosphorylation, and the activated cdc25C can remove the inhibitory tyrosine phosphorylation on cdc2, leading to its activation. CaMK II phosphorylated inactive cdc25C and marginally increased its activity, suggesting that CaMK II may be one of the relevant cdc25C kinase in cells [60,61]. To date, CaMK homologs have been successfully isolated from *Maluspumila*, *Liliumbrownie*, and *Nicotianatabacum* [62,63,64]. In maize, the accumulation of two CaMK homologs of MCK1 and MCK2 were identified and validated experimentally, particularly in plant parts undergoing rapid growth, such as the apical meristem, root cap, and flower primordium [65]. In animals, researchers have found that CaMK II could promote cell proliferation and growth by facilitating the replication of the centriole. Simultaneously, the G2 phase in the cell cycle could be arrested by the CaMK II inhibitor [55].

Therefore, we speculated that CaMK II may play a major role in regulating the cell cycle and rapid growth of single-celled dinoflagellates in the log growth phase. In *A. pacificum*, 2 cdc25 and 10 cdc2 were found in the transcriptome data (SRX368254), which indicate similar mechanisms in cell cycle control compared with animals and higher plants. The expression of CaMK2B was up regulated in LOG and HPM compared with LAG from RNA-seq and RT-qPCR (Figure 5). CaM was shown to interact with CaMK II, which provided evidence of its regulatory function in controlling cell cycle.

STK1 has been shown to participate in many biological processes, such as the cell cycle and apoptosis. It is conserved throughout phylogeny with hybridizing sequences being detected in DNA from mammals, amphibians, insects, and yeast. The expression of the STK1 gene is associated with cell proliferation in mouse cells. In the S phase, the mRNA level of STK1 begins to increase and then gradually decreases from the M to G1 phase. In addition, STK1 can interact with cdc37, forming a complex with CDK4 to control the cell cycle (G1/S transition) [66]. cdc37 works as a co-chaperone, targeting a variety of protein kinases for recruitment to the heat shock protein 90 (Hsp90) [67]. In addition, according to the transcriptome data of *A. pacificum*, there are two and 16 unigenes encoding cdc37 and Hsp90, respectively, which indicates that the cdc37-Hsp90 complex may play important roles in the cell cycle and signal transduction in *A. pacificum*. The expression of the STK gene was significantly up-regulated under LOG and HPM compared with LAG based on transcriptome data (SRX368254), which was consistent with the results of the 2-D analysis and confirmed by qPCR, suggesting that STK participate in *A. pacificum* cell cycle regulation (Figure 5).

### 3.3. Summary of CaM Interacting Proteins

**Figure 6:**
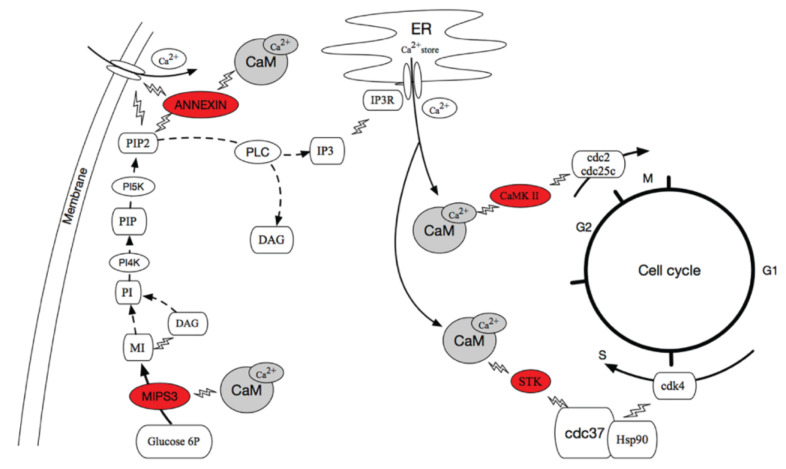
A network of proteins interacting with CaM and their target proteins. The rounded red rectangle represents the proteins identified in this study. The lighting symbols show the interactions between proteins. The solid lines show the processes were adapted from references in discussion 3.1 and 3.2.

We proposed a signaling pathyway in which CaM and its interactive proteins mediate Ca^2+^ transport and cell cycle in *A. pacificum*. As shown in Figure 6. We identified two proteins involved in the Ca^2+^ transport channel: MIPS3 and annexin. During the transport of Ca^2+^, the up-regulated MIPS3 of *A. pacificum* induced by HPM catalyzed the transformation of D-glucose 6-phosphate to 1D-*myo*-inositol 3-phosphate (MI), which provides an abundant supply of PIP2 precursors. The product of the PLC catalyzation of PIP2 was IP3. Next, IP3 entered the cytoplasm and activated IP3 receptors (IP3R) on the smooth endoplasmic reticulum (ER), which opened Ca^2+^ channels on the smooth ER. Meanwhile, the binding of annexin and PIP2 promoted membrane permeability to Ca^2+^, allowing Ca^2+^ to be transported into the cytosol, which provided sufficient intracellular Ca^2+^ to activate the Ca^2+^-binding and Ca^2+^-dependent proteins. After binding with Ca^2+^, CaM was activated to regulate downstream enzymes. Finally, MIPS3 and annexin regulated the process of cell cycle and proliferation.

Two proteins involved in the cell cycle were also identified: STK and CaMK II. During the transition from the G1 phase to the S phase in the cell cycle, STK interacted with cdc37 and Hsp90 to form a complex with CDK4 and this complex controlled the G1/S transition. Meanwhile CaMK II was activated by Ca^2+^/CaM, which accelerated the G2/M transition through interaction with cdc25c and cdc2. STK and CaMK2B of *A. pacificum* were up regulated under LOG and HPM compared with LAG, which accelerated the process of cell cycle through above ways.

CaM and its interactive proteins in *A. pacificum* may also play an important role in other regulation aspects, such as methylation of DNA and toxin precursors, because CaM has a wide range of biological functions and participates in organism growth and development and response to various stresses [68]. The properties and mechanisms need further investigation.

## 4. Materials and Methods

### 4.1. Sample Preparation and Collection

*Alexandrium pacificum* was obtained from the Key Laboratory of Marine Genetics and Breeding, Ministry of Education of China, Ocean University of China. Stock cultures of *A. pacificum* were grown at 20 ± 1 °C under a photon flux density of 30 μmol m^−2^ s^−l^ and a light: dark photo cycle of 12:12 h. Cultures were maintained in silicate-free f/2 medium [69].

**Figure 7:**
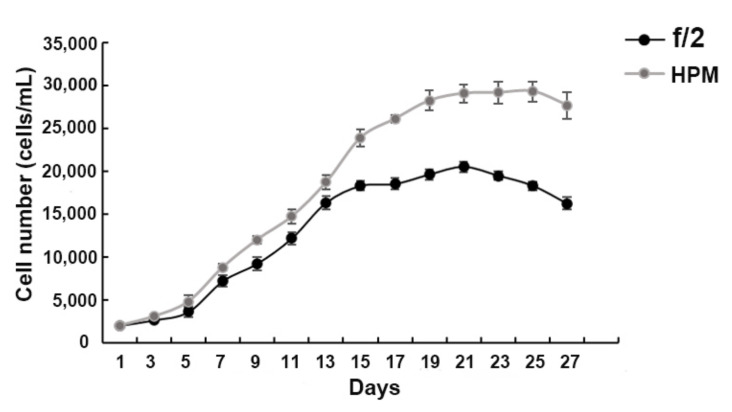
Growth curve of *A. pacificum* under f/2 and HPM. LAG phase: 1–4 days; LOG phase: 5–15 days; stationary phase: 15–22 days; decline phase: after 23 days.

Before the study, we cultured the algae in normal f/2 (natural seawater with f/2 medium) and HPM (NaH_2_PO_4_·2H_2_O and MnCl_2_·4H_2_O in f/2 medium were supplied at a final concentration of 0.144 mmol/L and 2.730 μmol/L) under laboratory conditions for 27 days. The number of algae was counted every two days using a Sedgwick counting box, and the growth curve was plotted (Figure 7). The HPM condition was chosen because phosphate and manganese have been reported to be involved in promoting cell growth [15]. Compared with the LAG and LOG, the algae in HPM are considered as in explosive growth.

The synchronized cells (placed in the dark for 48 h) were inoculated into three cultivating conditions at an initial concentration of 2 × 10^6^ cells/L for different treatments [70]. Finally, the samples were collected at LAG (natural seawater with f/2 medium; lag phase; 3 days), LOG (natural seawater with f/2 medium; log phase; 12 days), and HPM (NaH_2_PO_4_·2H_2_O and MnCl_2_·4H_2_O in f/2 medium were supplied at a final concentration of 0.144 mmol/L and 2.730 μmol/L, respectively; log phase; 12 days) conditions and frozen in liquid N2 for RNA isolation and RT-qPCR.

To reduce bacterial contamination during cell collections, every 100 mL of cultures was filtered through a 10-μm pore-sized bolting-silk, rinsed with 300 mL of sterile f/2 medium and subjected to the following treatments. The washed cells were suspended in 50 mL sterile f/2 medium containing 0.05% Tween-80 and 0.01 M ethylenediaminetetraacetic acid (EDTA; at 20 °C for 30 min), followed by ultrasonication (50 W; ultrasonic treatment time of 5 s with an interval of 5 s) for 1 min. Lysozyme (0.5 mg/mL, 20 °C for 10 min) and sodium dodecyl sulfate (SDS) (0.25%, 20 °C for 10 min) were then added. The cells were washed three times with sterile f/2 medium to remove these reagents. The final cultures were stained with acridine orange (0.01%) for 1 or 2 min, then observed using epifluorescence microscopy (Nikon ECLIPSE 80i, Japan), only the axenic samples were harvested and used for the following experiments. Each sample contained 5 × 10^6^ cells that were collected by centrifugation for 6 min at 4000× *g*, and then the cell pellets were transferred to a 1.5-mL eppendorf tubes for protein extraction.

### 4.2. RNA Extraction and First-Strand cDNA Synthesis

Total RNA of the samples was extracted following the manufacturer’s instructions using RNAiso Plus (TaKaRa, Dalian, China). The quality of RNA samples was measured by gel electrophoresis (1.5%) and a UV spectrophotometer (Tiannon, Shanghai, China). Only integrated samples and those with a ratio of absorbance at 260 and 280 nm between 1.9 and 2.1 were kept for further experiment. Following this, 1 µg of total RNA was used to synthesize first-strand cDNA according to the procedure of the PrimeScript™ RT reagent kit with gDNA Eraser (TaKaRa). The synthesis efficiency of every synthesized cDNA was tested by house-keeping gene amplification.

### 4.3. The cDNA Isolation and Sequencing

The sequences of the genes encoding CaM and the identified differentially expressed proteins were obtained from transcriptome data (SRX368254, https://www.ncbi.nlm.nih.gov/sra/SRX368254 (accessed on 22 July 2015)), and all the sequences also can be found in the Supplementary Material. The primers were designed by Primer Premier 5.0 for amplification by PCR (Table 2).

The complete cDNA sequence of CaM and the partial sequences of CAMK2B, STK, MIPS3 and ANNEXIN were amplified using the primers listed in Table 2. The PCR cycling parameters were as follows: one cycle at 94 °C for 5 min, 30 cycles at 94 °C for 30 s, 60 °C for 1 min, 72 °C for 1 min, and one cycle at 72 °C for 10 min. The PCR product was purified by TIANgel Midi Purification Kit (TIANGEN, Beijing, China) according to the manufacturer’s instructions. The concentration and purity of the obtained product was checked by gel electrophoresis (2.0%) and a UV spectrophotometer. After PCR amplification, the gene sequences were cloned into pMD18T (TaKaRa, Dalian, China) according to the manufacturer’s instructions. The sequences were then sent for DNA sequencing and analyzed using the Basic Local Alignment Search Tool of the National Center for Biotechnology Information.

### 4.4. Construction of Recombinant Plasmids

**Figure 8:**
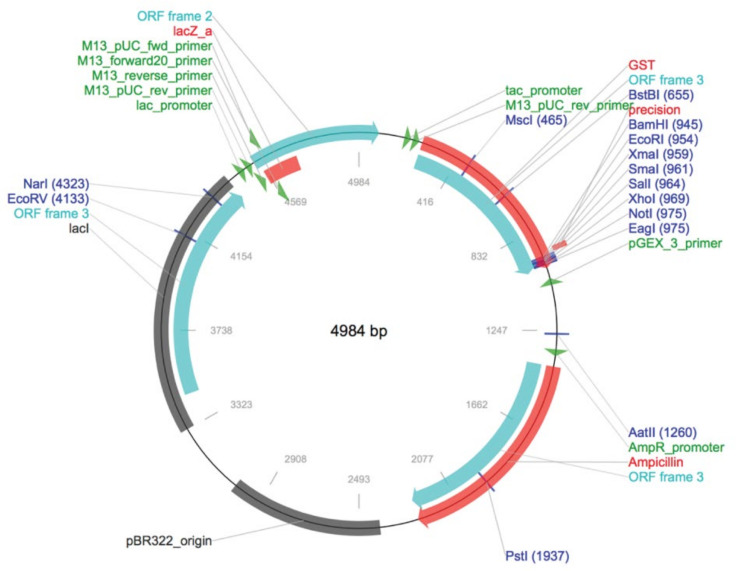
Plasmid profile of PGEX-6P-1.

The PMD18T-CaM plasmid was transformed into *E. coli* DH5α competent cells. The positive colonies carrying the target gene were selected by using fresh lysogeny broth agar plates supplemented with 100 mg/mL of ampicillin after growing for 12 h at 37 °C; PMD18T-CaM was then purified using the Plasmid Mini Kit (Omega, Guangzhou, China). The obtained plasmid and PGEX-6p-1 harboring glutathione S-transferases (GST) tag, which could be used for protein purification; (Figure 8) were digested with *Bam* HI/*Sal* I (TaKaRa) and connected by T4 DNA ligase (TaKaRa) to create recombinant plasmids which were confirmed by PCR and *Bam* HI/*Sal* I digestion and then sequenced. The correct recombinant prokaryotic expression plasmids were named as PGEX-CaM.

### 4.5. Prokaryotic Expression and Purification of GST-CaM Fusion Protein

The recombinant plasmids were transformed into *E. coli* BL21 (DE3) (TransGen Biotech, Beijing, China). Freshly transformed bacteria were inoculated into lysogeny broth medium (100 mg/mL of ampicillin) and grown at 37 °C with continuous shaking at 120 rpm until the absorbance at 600 nm reached 0.6. The GST-CaM fusion protein was expressed under the induction of isopropyl-1-thio-β-D-galactopyranoside (IPTG). The expression conditions were optimized with 1 mM IPTG, and an 8 h induction time at 28 °C was conducted. The pellet was then resuspended in lysis buffer, and the bacterium were disrupted using sonication (a total of 2 min with short pulses of 5 s) on ice. The cell pellet was washed in phosphate buffered saline Tween-20 (PBST; 0.1% Tween-20) and centrifuged at 12,000× *g* for 10 min at 4 °C. The supernatant was collected, and the pellet was analyzed by SDS-polyacrylamide gel electrophoresis (SDS-PAGE).

For purification of the soluble GST-CaM fusion protein, large scale BL21 (DE3) cells were cultured and harvested again. Soluble and insoluble fractions were separated by centrifugation at 12,000× *g* for 15 min at 4 °C. The supernatant was applied to glutathione-sepharose 4B resin and washed by glutathione buffer (50 mM Tris-base, 10 mM reduced glutathione, pH 6.0). The total protein concentration in the supernatant was determined by using a bicinchoninic acid protein assay kit (Sangon Biotech, Shanghai, China) according to the manufacturer’s instructions. The sample was then analyzed by SDS-PAGE.

### 4.6. Determination of GST-CaM Specificity

The purified GST-CaM fusion protein was subjected to 12% SDS-PAGE and transferred to a nitrocellulose membrane. The membrane was blocked at 37 °C for 1 h with 5% nonfat milk in PBST (0.1% Tween-20), followed by incubation with the anti-GST antibody (1:400 dilution, Sangon Biotech) at 4 °C overnight. After washing three times with PBST (containing 0.1% Tween-20), the membrane was incubated for 2 h at 37 °C with horseradish peroxidase conjugated goat anti-rabbit IgG (1:2000 dilution, Sangon Biotech). The membrane was then rinsed three times with PBST, and the protein bands were visualized using an enhanced chemiluminescence protocol and the western blotting detection system (Tiannon, Shanghai, China).

### 4.7. Protein Extraction

The algae were collected under LAG and HPM, and 1 mL Trizol reagent was added to the cell pellet after which it was subjected to sonication (2 min with short pulses of 5–10 s) on ice. Subsequently, 200 μL of chloroform was added to the cell lysate before vortexing for 15 s. The mixture was allowed to stand for 5 min at 25 °C before being centrifuged at 12,000× *g* for 15 min at 4 °C. The top colorless layer was removed, then 300 μL of ethanol was added to resuspend the reddish bottom layer, and the mixture was centrifuged at 4000× *g* for 5 min at 4 °C. The supernatant was transferred to a new tube, and 1.5 mL of isopropanol was added. The mixture was allowed to stand for at least 40 min for precipitation of proteins at 4 °C. It was then centrifuged at 12,000× *g* for 10 min at 4 °C. The pellet obtained was briefly washed with 95% ethanol before air drying.

### 4.8. The 2-D Analysis

Rehydration buffer containing 9 M urea (Bio-Rad, Hercules, CA, USA), 4% 3-[(3-cholamidopropyl) dimethylammonio]-1-propanesulfonate CHAPS (Bio-Rad), 40 mM dithiothreitol (DTT; Bio-Rad), and 2% immobilized pH gradient (IPG) buffer (GE Healthcare Life Sciences, USA) was added to solubilize the protein pellet. The solution was centrifuged at 12,000× *g* for 30 min at 16 °C, and the supernatant was collected for 2-D analysis.

Protein quantification was performed using the PlusOneTM 2-D Quant kit (GE Healthcare Life Sciences). Each sample containing 50 μg of protein mixed with a hydration solution was added to the focus plate. The IPG strips were rehydrated using rehydration buffer containing 8 M urea, 2% 3-[(3-holamidopropyl) dimethylammonio] -1-propanesulfonate, 15 mM DTT, and 0.5% IPG buffer. After rehydration, IEF was performed using the IPGphor 3 (GE Healthcare Life Sciences). Voltage control was performed using the following procedures: 300 V 2 h; 500 V 2 h; 1000 V 2 h; 5000 V 1 h; 8000 V 10 h. After the first dimension, each strip was equilibrated with approximately 10 mL of equilibration buffer (50 mM Tris pH 8.8, 6 M urea, 30% glycerol, 2% SDS, and 1% DTT) for 15 min. The gel strip was then equilibrated in fresh equilibration buffer containing 1% iodoacetamide (instead of DTT) for another 15 min. The second-dimension SDS/PAGE was performed using 12.5% polyacrylamide gel. After electrophoresis, the gel was used for far western blot analysis.

### 4.9. Far Western Blot

Far western blot was used to determine proteins that interacted with CaM [71]. Proteins extracted from the LAG and HPM of *A. pacificum* after 2-D analysis were transferred to a nitrocellulose membrane, the membrane was washed with 1×phosphate buffer solution to remove SDS. The proteins bound to the membrane were refolded by incubation in 6 M and then 3 M Guanidine-HCl in Tris buffered saline solution with Tween-20 (TBST; 10% glycerol, 100 mM NaCl, 20 mM Tris, 0.5 mM EDTA, 1 mM DTT, and 0.1% Tween-20) supplemented with 2% skimmed milk powder for 30 min at 25 °C. The blot was then washed with 1 M, then 0.1 M guanidine-HCl in TBST buffer supplemented with 2% milk powder for 30 min at 4 °C. The membrane was rinsed in TBST buffer and blocked in TBST buffer containing 2% skimmed milk at 4 °C overnight. The membrane was incubated with purified GST-CaM fusion protein and GST tag (work as control) at 4 °C for 2 h, respectively. Then, the membrane was washed three times with TBST buffer for 10 min at 25°C and further incubated with the anti-GST antibody in TBST buffer with 2% milk. After the membrane was washed three times with TBST buffer for 10 min at 25 °C, it was then incubated for 1 h with the secondary antibody HRP-conjugated goat anti-rabbit IgG (Sangon Biotech). The protein bands were then visualized with an enhanced chemiluminescence protocol and the western blotting detection system (Tiannon).

### 4.10. MALDI-TOF MS Analysis

After 2-D and far western blot analyses, the differentially expressed protein spots were identified by PDQuest software and digested in gel with trypsin (10 ng/μL in 25 mM ammonium bicarbonate) at 37 °C for 12 h. Protein identification was conducted using an AB SCIEX MALDI TOF-TOF™ 5800 Analyzer (AB SCIEX, Foster City, CA, USA).

All database searching was fulfilled using GPS Explorer™ software (version 3.6, AB SCIEX) running a mascot search algorithm (v2.2, Matrix Science, London, UK) for protein identification. Results with confidence interval values greater than 95% were considered as a positive identification.

### 4.11. Quantitative PCR to Determine the Gene Expression of Differentially Expressed Protein

The qPCR was performed with Light- Cycle^®^ 480 Real-Time PCR System with SYBR Green Real time PCR Master Mix (Applied Biosystems Ltd., Shanghai, China). Standard curves of each gene in every qPCR run were produced through serially diluted plasmids containing the gene of qPCR. The plasmids were diluted 10-fold six times (from 10^−1^ to 10^−6^). The program of reverse transcription was 2 min at 50 °C, 2 min at 95 °C followed by 40 repeats of 15 s at 95 °C, 1 min at 60 °C, one cycle of 15 s at 95 °C, 1 min at 60 °C, one cycle of 15 s at 95 °C, and 15 s at 60 °C. Actin and GAPDH were used as normalization genes, which have been broadly used for various marine algae, including *Alexandrium*. The 2^−ΔΔCt^ method was used to assess gene expression [72]. The mean fold change was calculated for each experiment, and a mean >2 or <0.5 was considered significant [73].

## 5. Conclusions

Our study identified four proteins interacting with calmodulin in *Alexandrium pacificum*, including MIPS3, annexin, STK, and CaMK II. The gene expression levels of calmodulin interacting proteins were up-regulated under LOG and HPM conditions, especially HPM condition, which was the period of explosive growth of *Alexandrium pacificum*. Based on the functional analysis of calmodulin and its interacting proteins, we hypothesized a schematic model to illustrate that calmodulin and its interacting proteins participate in the explosive growth and proliferation through Ca^2+^ transport and cell cycle pathway in *Alexandrium pacificum*. Overall, the results of this study open new insights into the functioning of CaM and interacting proteins for cell cycle regulation in *Alexandrium pacificum*.

## Figures and Tables

**Table 1:** Differential expression of proteins between HPM and LAG revealed by 2-D analysis.

Spot ID	Accession Number	Protein Score	Protein Score CI%	Peptide Count	MW/pI	Protein Description	HPM vs. LAG	
							Fold Change	*p*-Value
1	GL433858.1	145	100	3	74.39/5.81	Calcium/calmodulin-dependent protein kinase 2B		
3	CAA48538.1	121	99,248	4	47.30/6.79	Serine/threonine kinase		
6	NM001003039.1	116	99,997	5	80.08/4.12	Annexin A4	2.5	0.003
8	XM001703196.1	150	100	3	80.52/5.54	Inositol-3-phosphate synthase	2.2	0.007

**Table 2:** Primers used in this study and their characteristics.

Name	Sequence 5′→3′	Application	Size (bp)
cam-f	CGGGATCCATGGCTGACCAGCTCACG	Complete cDNA sequence of CaM cloning	450
cam-r	CACGCGTCGACTCACTTTGCCATCATCATCTTCAC		
CAMK2B-f	AAAATCTGTGATCCACACTTGACT	Ca^2+^/calmodulin-dependent protein kinase (CAMK2B) cloning and qPCR	114
CAMK2B-r	CTTACAGTTCTTCCCCAGGACATT		
STK-f	GCACTTGCCGACACGCTTACAT	Serine/threonine-protein kinase (STK) cloning and qPCR	170
STK-r	GTGAGCCGACTGGGTTTTCCTT		
MIPS3-f	CAACCACTTGGGGAACAATGATGG	*Myo*-inositol-3-phosphate synthase(MIPS3) cloning and qPCR	138
MIPS3-r	AATAGTGTTCGTTCCCCCCATAAC		
ANNEXIN-f	CCTCTCTTGGTCGCTTTACTTGTC	ANNEXIN cloning and qPCR	182
ANNEXIN-r	TGACAACTTGACGCTTTGGACC		

## Data Availability

The data presented in this study are available in the article.

## Supplementary Materials

The following are available online at: https://www.mdpi.com/article/10.3390/ijms23010145/s1.
