# Supplementary material for: Calmodulin and Its Interactive Proteins Participate in Regulating the Explosive Growth of Alexandrium pacificum (Dinoflagellate)
Source: Int J Mol Sci. 2021 Dec 23;23(1):145. doi: 10.3390/ijms23010145 (PMC8745774; doi:10.3390/ijms23010145)
Supplement: Supplementary file 1 [file ijms-23-00145-s001.zip › ijms-1475184-supplementary.pdf]

MIPS3：

Algae_064-3_Unigene_BMK.18692

GAGAGAGAAAGAGAGAGAAAGAGAGAGAAATAGTTCGAGTGAGTTGGCAACCACCCTTTTTTT
CATTTTTTGAAAAAGATCCGAAAAAAAGGAGAGAGAGAGAGAGAGAAAATGTTTATTGAGAGC
TTCAAAGTAGAGAGCCCAAACGTGAAGTACACAGAGAATGAGATTGAATCGGTGTACAACTAC
GACACCACCGAGTTGGTTCATGAGAATCGCAAAGATGCTGGTGGTTATCAATGGATCGTTAAGC
CCAAGACCGTTCAGTATCACTTCAAGACTGACACTCGTGTCCCCAAACTCGGGGTGATGCTGGT
TGGATGGGGTGGAAATAACGGCTCCACCCTCACAGGTGGTGTGATCGCCAACCGCGAGGGAAT
ATCTTGGGCAACCAAGGACAAGATCCAGCAAGCGAACTATTTTGGTTCGTTGACCCAGGCTTCT
TCAATTCGGGTCGGGTCTTTTAATGGAGAGGAGATTTATGCTCCTTTCAAGAGCTTGCTCCCTAT
GGTGAACCCAGATGACGTAGTGTTTGGGGGTTGGGACATAAGCGACATGAACCTGGCTGATGC
AATGACCAGGGCTAGGGTCTTTGACATTGATCTTCAAAAGCAGCTCAGGCCTTACATGGAGCAC
ATGGTTCCCCTCCCTGGTATTTACGACCCTGATTTCATCGCTGCGAACCAAGGCTCCCGAGCCA
ACAACGTCATTAAGGGCACCAAGAAGGAACAAGTTGAGCGAGTCATTAAAGACATTAGGGAGT
TCAAGGAGAAGAACAAAGTGGACAAGGTGGTGGTGCTATGGACTGGCAACACAGAGAGGTAC
AGCAATGTGGTGGTCGGGCTCAACGACACCATGGAGAACCTGTTGGCATCTCTGGAGAAAAAC
GAGTCGGAGATTTCTCCATCCACTTCGTATGCCTTGGCATGTATCGAAGAAAATATTCCCTTCAT
CAATGGTAGCCCTCAGAACACCTTTGTGCCAGGGCTCATTGATCTGGCTATCAAGAAGAACAGT
TTGATTGGTGGTGATGACTTCAAGAGTGGTCAGACCAAGATGAAATCTGTCCTCGTCGACTTCCT
CGTTGGGGCTGGTATTAAGCCAACATCCATAGTGAGCTACAACCACTTGGGGAACAATGATGG
GATGAACCTGTCAGCACCTCAAACTTTCAGGTCCAAGGAGATTTCTAAGAGCAACGTGGTCGAT
GACATGGTTGCCAGCAACGGCATCCTCTATGAGCCGGGCGAACACCCTGATCATGTTGTGGTCA
TCAAGTATGTGCCATATGTGGGAGACAGCAAGAGAGCCATGGATGAGTACACTTCGGAAATATT
TATGGGGGGAACGAACACTATTGTCATGCACAACACCTGTGAGGACTCTCTCTTGGCTGCCCCC
ATCATCCTGGACTTGGTCCTCCTTGCTGAACTAAGCACCCGCATTCAGCTGAAGGCTGAGGAAG
AGGACAAGTTCCACTCCTTCCATCCGGTCGCTACCATCCTGAGTTACCTCACCAAGGCCCCTCTT
GTTCCACCAGGCACTCCAGTGGTAAATGCACTTTCGAAGCAGAGGGCTATGCTGGAGAACATA
CTGAGGGCTTGTGTTGGTTTGGCTCCTGAGAACAACATGATCCTCGAATACAAGTGAGGATGAA
GTCACAAGAGCCACACCACAAGGCAACCCTATCCGAGCTGTTTTATCTTTGCGTTTGCTATTGTT
GTGGCGT

CAMK2B

Algae_064-1_Unigene_BMK.89842

GCGGCCGGCAGTGGCTCTCTCGCGTTCGCGTTTCCGAGTTTGGTGGTGCGATTCAAATAATCGT
GAATAACCTCGGTATGGCCGTGCCAACGGCTTGCACGCGGTTCAGTGACAACTACGACTTGAA
GGAGGAACTCGGCAAAGGTGCCTTCTCCGTGGTTCGACGATGCGTACAAAAGAGCACCGGTCA
CGAGTTTGCTGCAAAAATCATCAACACCAAAAAGCTGTCGAACAGAGATTTCCAAAAACTTGAG
CGTGAAGCGCGAATATGTAGAAAACTGCAGCATCCAAATATTGTGAGATTGCACGACAGTATAC
AAGAAGAGAATTTCCATTATCTCGTCTTTGATCTAGTCACCGGCGGGGAACTGTTCGAAGACAT
CGTTGCTCGGGAATTCTACAGCGAGGCCGACGCCTCGCACTGTATTCAACAAATCCTGGAAAGC
GTTCACCACTGCCACCATAATGGAGTTGTGCATCGGGATTTGAAACCCGAAAATTTACTCCTCG
CGAGCAAGGCGAAAGGGGCGGCTGTGAAATTAGCGGACTTTGGCTTAGCAATTGAGGTGCAGG
GTGAAGCACAGGCATGGTATGGTTTCGCTGGAACGCCCGGCTATCTCAGCCCGGAGGTACTGA
AGAAGGAACCGTACGGAAAGCCAGTCGACATATGGGCGTGCGGTGTCATCTTGTATATCCTTCT
CGTCGGCTATCCGCCGTTCTGGGACGAGGATCAACATCGGCTCTATGCACAGATCAAGGCCGG

ATCGTACGACTATCCGAGTCCAGAATGGGACACCGTCACGCCGGAAGCCAAGAATCTTATCAA
TCAAATGCTGACGGTGAATCCAGGAAAGAGGATCACTGCCAGCGAGGCATTGAAGCATCCATG
GATCTGCCAACGTGAGCGTGTTGCATCTGTGGTGCATAGACAAGAAACCGTGGACTGCTTGAAG
AAATTCAACGCAAGGCGCAAATTAAAGGGCGCTATACTGACAACCATGTTGGCGACGCGGAAC
TTTTCCAGTAAGTATGATGCACAGGGTCGAAGCATCATCACGAAGAAGGGCGATGGCTCTCAA
GTGAAGGAATCTACCGATAGCAGCACAACGATCGAAGATGATGACGTTAAAGAGGATAAGAAG
GGCGGCGTCGACCGGAGCAGCACGGTCATTGCCAAAGAACCCGAAGCGCGCCGGCAGGAAAT
CATCAAGATGACCGAACAATTGATCGAAAGCATCAACACTGGAGATTTCGAGGCATACACGAA
AATCTGTGATCCACACTTGACTGCGTTCGAGCCGGAGGCTCTAGGTAATTTAGTCGAGGGAATG
GATTTTCACAAATTTTACTTTGATAATGTCCTGGGGAAGAACTGTAAGGCCGTCAATACAACAAT
CTTGAATCCTCACGTCCACTTGCTCGGCGAAGATGCCGCTTGCATCGCGTACGTTAGGCTGACG
CAATACATGGACAAACAAGGTGTAGCTCACACCCAGCAGAGCGAGGAGAGTCGTGTATGGCAC
AAGAAGGACAACAAGTGGCAGAATGTGCATTTCCACCGAAGCGCGGTGACGGGCCCGTCACC
GTTCTCTTTCAACCACAAATAAATGGACCGAGAGGACTATTCCGCTGCTGCGCTCGGTGGACGC
TCATCATCTCCCGAGAGAGCGCGAATGAATTAACGAACGTTAGGAA

ANNEXIN

>Algae_064-2_Unigene_BMK.54942

CCCTTGACGTAGCGCTCCACTGGCATGTCCATCAGGGAAAGCATCGTGGTCTTCCAGTAGTTGG
AGAAGGAGAACCAGGACTTGCCCTCCTCGCACTTCTTGGCAATTTCGTCTCGGAGCGAGGCGC
CGTAGACCGTCTCGTAGAGCCCCAAGTGCCGCTTGATAGCGACAGCGGGCATGGAGACGAGA
ACCTCCTTCACCGTGTCGATGTCGCGGACGGCGTCGAGGGTCCTCTGTGCCCAGTAGAGGGGC
ATGAGGTCGAGATCGTCCTCGCTGTTTCGCCCGGCAATCTCCAGCAGCGTCCTCTTGTAGTCGC
CGCTGCAGTCGCCGTCGATCCAGTCCTCGAGCATCTTGCCGTTCATGGCAAAGAAGGCCCTGCG
CACCTCCAGCATGTCGTCCTTCGCGAGCACCATGAAGTTGATGAGCAACTGGTCCGAAGTGCCC
AGACCTTTCATGGCACCCGTGAGGGCCTCGGCCCAGACGACGGGCGCCGGCTTAACCAGGCA
GCAAAGGGCCTTCTCATAGTCACCGCTGGTGTCGCTCTTGATGTCTTTGATGAGGTCGCGCCCTT
TGAACTCTTTGGCGTAGGTCTTGATCAGGGCAGCCTTCTTGAAGTCTTCCAGGTGCACGAGGCA
GGTGATCAGCCCGGTGTCGTCCGTGCCCCAGCCGGCGATGCAGTCGCGCACGGAGTGGGCAA
GCTGGACCATGGGGTGGCGGATGCAGCCCAGCAGCGTGGCCTGAAAGTTTCCGCTGGTCTCAC
TCTTCACCCAACCAAGCAAGTCCCTGTCATAGAGCTCCTGGAAGCGCTTGTTCACGTCCTCCATC
TGCTTCGAGGTCTTGTTGCAGATCATCTGTATGAGCGCCGCCTGGTCGGTGCCCCAGCCGTTCA
TGGCCTCCTCCATGGCGGCGCAGTCCGCGTCGAGGTCGAAGGGGGCCTCGGAGGCCTCGAGC
ATGCACTTCAGGGACTTCTTGAAGTCGCCGCTGGTCTCGCTCTCGATGTGCTCGATCAGGTCTCT
GCCGAACATGCGCTCGTAGGCCTCCTTCACTTGCCGGCGCTCGCCGAAGTCCAGCGCGCAGAT
CACGCGGATGAGCTGGTCCTCGGAGGTCCCCAGGCCCACCATCGCCCGGTTCAGGATCGTGGC
CCGGCAGTCCTCGGGGGCCATGGCCTGCCAGACCATGACCTTCTGGTAACTGCAAGAGGTCTC
ACTCTGGATGTGATCAAGGAGATTCTTGCCCGTCGTCTCCATATACTGCTGGAAGATCGGCACC
CGCAGGCGCTCCGGCATGGTGCAGACGAGCGCGGTGAGCGTGCCCTCGTCGGTGCCCCACCC
CTTCATGGCATCGTTCAGGCAGCGCACCCAGAGGTCGACCTCGGGCTCAAGCAGGGCAGCCAT
GGCGTGCTTGAAGTAGTTGGAGCGGATCCAGCCATCCTGCTCCGAGGCCTTCGTCACGATCTCC
ACAGCGACGTTGCCAGCGTGCAGCTCGTCGCTGTACTTGGCAACCATGTCCTGCGTGGTCTTGC
AGCCGATCGCGGCGAGCACCTCGATGAGAGCCTCCTCCTCGTGCAGCGCGTCGTTGCAGCGCT
GCGCGATGTTGCCTAGGACCTCCGCGTGCTGCAGCTGCTCCTCCGGCATACCTCCTCCATCTGC
CCACGCGGCAAACGACGGGAAGGGGATGTTGCCAGTGTGCGCCGGGTCGGCTGTGGCCATGA

TCTCGTCGATCTCGTTAGGCGACACTTCCTCGCCCCTGACCATGCAGATGAGCGCGTCCAGCTC
GGCCTTGCTGATGGAGCCATTGCCATCCAGGTCGAAGGCCTGGAACTGGAACCTCCTTGCCAG
CTCCGTCGTGGAGGACTCCAAGACGGCCTTCTTCAGGATCGTCATGGTCTGGGGCTCCCTGCGC
TGCCAGCGCTTGGCGTAGCGGTCCGTGCCCTCAAAGTACTTCTCCTCCACGTAGTTGTGCAGCC
AAGACTGCAGGTACACCGTATTCCGGTGCTTTAACGCCATGGGCCGACCTTGCTTGAAGAGGCC
GAAGAGCTGCCCCGGAGAGTTCTCATCCCTGTCCGCTAGTGCCAGAGGCTGGCTGTCTGTGGCT
GACTCGAGGTACTTGCCCGTCTCCACGTGCTCCAGGAGCACGTCGTCACCGTCCTGGAAGACG
CCGTTCGTGCCGTCCCTCCTCCAGCAGCGGAAG

STK：

>Algae_064-2_Unigene_BMK.28161
CTTCAAGGGGAGGCGGAGGTTCTCTGGCCAGCTGGTGGCGCTGAAGTTCATTTCGAAGAGGGG
AAAGCCTGAGAAGGAGCTTCAGAACTTGCGCTTGGAGATTGGGATCCTCAAGCGCTTGGACCA
CCCGAACATTATCCGGATGCTTGACTCCTTCGAGACGAGCACCGACTTCGTCGTGGTGACAGAG
TTTGCCTACGGTGAGCTCTACGAGATCTTCCAGGACGACAAGAATCTACCCGAGGACGAGGTC
AGGCACATCGCCAGGCAGTTGACCCAGGCACTCTACTACCTCCACTCGCAGAAGATCATACAC
CGTGATATGAAGCCGCAGAACGTCCTTGTCGGCGCCAACGACACCATCAAGCTCTGCGACTTTG
GCTTTGCGCGTGTCATGTCGTGCCAGACCACGGTGCTTACCTCCATCAAGGGCACGCCACTGTA
CATGGCTCCCGAGCTCGTGCAGGAGCGGCCGTACGATTGCAGTGCCGACTTGTGGCCCTTGGG
CGGGATCTGCTATGAGCTCTTCGTTGGTCAACCCCCCTTCTACACGAACTCTCTCATTTCGCTCA
TCCACCTCATCGTCGACAAGAAGGTGAGGTATCCGGAGAACATGTCGCCAGAGTTCAGGTCGTT
CCTTCAAGGCCTCTTACAGAAGAACCCCAAACAACGTTTGGGTT

CaM:

GCCATTTCCCTTGTGCCCACCAAGC**ATG**GCTGACCAGCTCACGGAGGAACAGATTGCCGAATTC
AAGGAGGCCTTCAGCTTGTTTGACAAGGACGGAGACGGCACCATCACGACGAAGGAGTTGGG
AACGGTGATGCGATCCCTCGGCCAGAACCCCACTGAGGCGGAGTTGCAGGATATGATCAACGA
GGTTGATGCTGATGGCAACGGGACTATTGACTTCCCTGAGTTCCTTTCCCTCATGGCCCGGAAG
ATGAAGGACACAGACACGGAGGAGGAGCTCATTGAGGCCTTCAAAGTGTTCGACCGTGATGGC
AATGGCTTCATCAGCGCTGCCGAACTGCGGCACGTCATGACCAACCTGGGCGAGAAACTCACA
GACGAGGAGGTTGATGAGATGATCAGGGAGGCCGATGTGGACGGAGACGGACAAATCAATTA
CGAGGAGTTCGTGAAGATGATGATGGCAAAG**TGA**TCAGAGCGGTAAAATACCGCAGGCGAGC
GCGGCTGAGTCTGAACTCATCATGGATCGCAAGCTAATTAGACAGAGATAATGCAAGTCTCTTG
ACCAAGGCAGAACAAAGAAAAAAAAAAAAAA
